# Identification of TIMP1-induced dysregulation of epithelial-mesenchymal transition as a key pathway in inflammatory bowel disease and small intestinal neuroendocrine tumors shared pathogenesis

**DOI:** 10.3389/fgene.2024.1376123

**Published:** 2024-08-21

**Authors:** Minh Tuan Tran

**Affiliations:** Indian Springs School, Pelham, AL, United States

**Keywords:** inflammatory bowel disease, small intestinal neuroendocrine tumors, differentially expressed genes, epithelial-mesenchymal transition, warburg effect, intestinal fibrosis

## Abstract

Inflammatory Bowel Disease (IBD) is believed to be a risk factor for Small Intestinal Neuroendocrine Tumors (SI-NET) development; however, the molecular relationship between IBD and SI-NET has yet to be elucidated. In this study, we use a systems biology approach to uncover such relationships. We identified a more similar transcriptomic-wide expression pattern between Crohn’s Disease (CD) and SI-NET whereas a higher proportion of overlapping dysregulated genes between Ulcerative Colitis (UC) and SI-NET. Enrichment analysis indicates that extracellular matrix remodeling, particularly in epithelial-mesenchymal transition and intestinal fibrosis mediated by TIMP1, is the most significantly dysregulated pathway among upregulated genes shared between both IBD subtypes and SI-NET. However, this remodeling occurs through distinct regulatory molecular mechanisms unique to each IBD subtype. Specifically, myofibroblast activation in CD and SI-NET is mediated through IL-6 and ciliary-dependent signaling pathways. Contrarily, in UC and SI-NET, this phenomenon is mainly regulated through immune cells like macrophages and the NCAM signaling pathway, a potential gut-brain axis in the context of these two diseases. In both IBD and SI-NET, intestinal fibrosis resulted in significant metabolic reprogramming of fatty acid and glucose to an inflammatory- and cancer-inducing state. This altered metabolic state, revealed through enrichment analysis of downregulated genes, showed dysfunctions in oxidative phosphorylation, gluconeogenesis, and glycogenesis, indicating a shift towards glycolysis. Also known as the Warburg effect, this glycolytic switch, in return, exacerbates fibrosis. Corresponding to enrichment analysis results, network construction and subsequent topological analysis pinpointed 7 protein complexes, 17 hub genes, 11 microRNA, and 1 transcription factor related to extracellular matrix accumulation and metabolic reprogramming that are candidate biomarkers in both IBD and SI-NET. Together, these biological pathways and candidate biomarkers may serve as potential therapeutic targets for these diseases.

## 1 Introduction

Inflammatory Bowel Disease (IBD), encompassing the two distinct phenotypes of Crohn’s Disease (CD) and Ulcerative Colitis (UC), are chronic inflammatory disorders of the gastrointestinal tract characterized by a dynamic interplay of genetic, environmental, immunological, and microbial factors (Zhang and Li, 2014). CD typically exhibits granulomatous inflammation in different parts of the gastrointestinal tract (Feuerstein and Cheifetz, 2017), whereas UC primarily affects mucosal layers of the colon (Ungaro et al., 2017). In both CD and UC, however, the chronic inflammatory condition displayed significantly increases the risk of developing various gastrointestinal malignancies, in which their relation with colorectal cancer is most extensively studied. Specifically, the inflammatory microenvironment drives genomic and epigenetic alterations, introduces oxygen-reactive species and cytokine mediators like the tumor necrosis factor-alpha (TNF-ɑ) or Interleukins-6 (ILs-6), and disrupts the intestinal microbiota homeostasis, all of which accelerates carcinogenesis (Axelrad et al., 2016).

Despite extensive efforts in deciphering the molecular mechanisms behind various IBD-associated neoplasms, the relationship between IBD and Small Intestinal Neuroendocrine Tumors (SI-NET), a malignancy of the specialized enterochromaffin cells found mainly in the distal small intestine, remains largely elusive (Gonzáles-Yovera et al., 2022). SI-NET is a rare cancer with growing prevalence, witnessing a 6.4-fold increased incidence rate between 1973 and 2012 (Yao et al., 2008). Recent efforts in uncovering SI-NET pathogenesis primarily lie in identifying genetic mutations (e.g., copy number variants in chromosome 18), implicating the role of micro-RNA and transcriptomes, and exploring several regulatory pathways (e.g., PI3K/Akt/mTOR and TGF-β pathways) (Xavier et al., 2016); however, impacts of other common carcinogenesis’ risk factors, particularly those above IBD-related such as immune dysregulation, environmental factors, or microbial compositions, are mostly unknown.

Currently, the linkage between the two diseases is most strongly implied through statistical analysis of disease incidences: a study by Yu et al. (2022) found a 3.5 and 2.3-fold increased risk of developing SI-NET secondary to CD and UC respectively. Microbiologically, the intestinal microbiota composition of IBD and SI-NET shares the depletion of *Faecalibacterium prausnitzii*; still, its influence on the disease phenotypes requires further investigations (Vitale et al., 2021). Immuno-wise, a possible linkage between IBD and SI-NET lies in the role of enterochromaffin cells, which are known to participate in gastrointestinal inflammation and hyperalgesia, proliferating in both inflamed and non-inflamed areas in IBD (O’Hara et al., 2004). Moreover, pro-inflammatory cytokines TNF-ɑ and IL-6 are found in gastroenteropancreatic neuroendocrine tumors, suggesting a role in tumor progression (Mahečić et al., 2020). Despite a more inhibitory immune microenvironment within SI-NET, high peri-tumoral immune activities, as observed in CD8^+^ cell populations, indicate potential immune involvement in carcinogenesis (Vesely et al., 2022).

This study, through comparison of IBD and SI-NET molecular profiles, aims to uncover 1) different relationships between SI-NET and the two different IBD phenotypes, 2) pro-inflammatory genes and proteins shared by IBD and SI-NET, particularly in SI-NET pathogenesis and tumor microenvironment, 3) diagnostic biomarkers and therapeutic targets in both diseases, and 4) possible biological pathways and molecular mechanisms that are both expressed in IBD highly immune-active and SI-NET immunosuppressive environment. We hypothesized that CD and SI-NET would have a stronger relationship than UC and SI-NET, considering the locations of inflammation, the shared immune cytokine mediators, and incidence rates. Moreover, these shared dysregulated genes between IBD and SI-NET would partake in SI-NET tumor microenvironment and tumor-stroma interactions exacerbated by inflammatory and cytokine-mediated responses.

## 2 Materials and methods

### 2.1 Data curation

Microarray datasets of patients with Crohn’s Disease (CD), Ulcerative Colitis (UC), and Small Intestinal Neuroendocrine Tumors (SI-NET) were curated from the Gene Expression Omnibus (GEO) database (Barrett et al., 2013), a freely-accessible repository of genomic data. Selected datasets include at least three mucosal biopsies of diseased and healthy control (HC) samples from the terminal ileum for CD and SI-NET and the colon for UC. Additionally, CD and UC samples must be taken from active inflammatory sites excluding ulcers, and SI-NET samples must be taken from well-differentiated primary tumors of enterochromaffin cells. Subsequently, three different datasets, GSE75214, GSE38607, and GSE65286, corresponding to CD, UC, and SI-NET respectively, were selected for further analysis.

### 2.2 Data normalization and preprocessing

Initially, raw data from GEO supplemental files was loaded into RStudio (version 2024.04.1) for quality control and normalization. Data from Affymetrix platforms were normalized with the Robust Multichip Average (RMA) method, which includes a three-step process of background correction, quantile normalization, and probe set summarization specialized for Affymetrix microarrays. RMA preprocessing was performed with the “*oligo*” (Carvalho and Irizarry, 2010) Bioconductor package (version 1.68.2). Similarly, for the Agilent one-color array platform (GSE65286), data was background corrected, quantile normalized, and log2 transformed with the “*limma*” package (version 3.60.3) (Ritchie et al., 2015). Moreover, quality control was performed with the “*arrayQualityMetrics*” package (version 3.60.0), which generated comprehensive assessments of individual array quality, array intensity distribution, variance mean dependence, and between-array comparison (Kauffmann et al., 2009). Finally, normalized expression matrices were subjected to prefiltering and probe-ID to gene conversions, in which multiple probes referring to the same gene were averaged while control and low-quality probes were disregarded.

### 2.3 Comparison of gene expression profiles of Crohn’s disease, ulcerative colitis, and small intestinal neuroendocrine tumors

The different relationships between gene expression levels of CD and SI-NET as compared to UC and SI-NET were assessed by analyzing the global expression patterns in each dataset as well as overlaps between differentially expressed genes (DEGs).

#### 2.3.1 Differential gene expression analysis, principal component analysis, and bidirectional hierarchical clustering

In Qlucore Omics Explorer version 3.9 (Qlucore AB, Lund, Sweden)., expression levels of diseased and HC samples were differentiated with the Welch *t*-test, an alternative for the Student’s *t*-test that is more suitable for smaller-sized and less uniformly distributed independent groups. Differentially expressed genes (DEGs) in all datasets were filtered with an adjusted *p*-value <0.01 and a│log2(Fold Change) > 1.5│. After identifying the DEGs, principal component analysis (PCA) was performed to visualize the separation between diseased and HC samples, both before and after differential gene expression analysis. Additionally, bidirectional hierarchical clustering was employed to examine expression patterns and identify distinct clusters of DEGs.

#### 2.3.2 Rank-rank hypergeometric overlap

The three unfiltered gene sets of CD, UC, and SI-NET from the Welch *t*-test were subjected to further downstream analysis with rank-rank hypergeometric overlap (RRHO), a threshold-free method that considers the global transcriptomes of each disease phenotype (Plaisier et al., 2010). RRHO treated the gene expression sets as per the hypergeometric distribution (i.e., Fisher’s one-tail exact test) and calculated whether the observed overlaps are statistically significant as compared to random chance. In this approach, gene lists of UC, CD, and SI-NET were ranked based on the recommended formula (Equation 1), with the most upregulated genes ranked at the top and the most downregulated genes at the bottom of the list:

$$rank=sign\left[\log_{2}\left(Fold\;change\right)\right]*-\log_{10}\left(unadjusted\;p-value\right)$$ (1)

The global transcriptomics analysis was performed with the RRHO R package (version 1.44.0), in which the relationships between two IBD phenotypes and SI-NET were analyzed with the “*RRHO*” function and the statistical significance of the difference between overlap was further determined with the “*RRHOComparision*” function. Next, the relationship strength between two ranked lists is assessed with Spearman’s ‘rho’ correlation, a substitute for Pearson’s correlation coefficient for non-parametric data. Finally, RRHO results were validated through gene set enrichment analysis (GSEA) Kolmogorov-Smirnov statistic-based procedure, which compares the enrichment of the two continuous ranked gene lists in one dimension (i.e., one varying threshold). That is, the ranked gene list of SI-NET is differentiated into upregulated and downregulated genes before comparison with the ranked gene lists of CD and UC.

#### 2.3.3 Jaccard similarity index of overlapping DEGs

The Venn diagram tool in Qlucore Omics Explorer was used to identify common DEGs (co-DEGs) between CD and SI-NET and UC and SI-NET respectively. DEGs are separated based on the “direction” of fold change, such that identified co-DEGs are commonly upregulated or downregulated in both disease states. Subsequently, the overlaps between CD and SI-NET versus the overlaps between UC and SI-NET were compared with the Jaccard Index (Equation 2), a mathematical measurement of the similarities of two different sets.

$$J\left(A,B\right)=\frac{a\cap b}{a\cup b}$$ (2)

The Jaccard similarity index accounts for the different in dataset sizes between CD, UC, and SI-NET. By comparing the list of DEGs in each cohort directly, we aimed to capture the more specific biological relationships between IBD and SI-NET.

### 2.4 Enrichment analysis of biological pathways and functional annotations

The lists of DEGs in CD, UC, and SI-NET as well as overlapping RRHO genes are subjected to downstream enrichment analysis to elucidate the biological mechanisms underlying changes in gene expression in each disease state. In detail, gene set enrichment analysis (GSEA), a commonly used algorithm to detect pathway-level changes, was used to determine whether genes within a biological pathway are consistently up-or downregulated in each condition. Complementing GSEA results, over-representation analysis (ORA) was performed to identify functional categories associated with sets of genes and, henceforth, provide insights into the biological and biochemical properties as well as cellular locations that characterize the gene sets.

#### 2.4.1 Gene set enrichment analysis

GSEA was performed in Qlucore Omics Explorer’s GSEA workbench and the Reactome package (c2.cp.reactome.v2023.2.Hs.symbol) curated from the Molecular Signature Database (MSigDb) (Subramanian et al., 2005; Liberzon et al., 2011). Currently, the Reactome package includes the greatest number of gene sets among Canonical Pathways in MSigDb, accounting for more specific biological processes. In addition to Reactome pathways, the immune gene sets (c7.immunesigdb.v2023.2.Hs.symbol) were also downloaded to further characterize the immunological landscape of SI-NET and elucidate potential overlapping pathways with IBD.

Considering that the number of samples analyzed in this study is limited and has a direct negative effect on the q-value false discovery rate (FDR) computed by GSEA (i.e., pathways with normalized enrichment score (NES) >│1.5│ and *p*-values <0.025 yet FDR >0.25), statistically significant biological pathways are determined as those with an NES > │1.5│ and a more relaxed FDR <0.45. Afterward, pathways were ranked based on NES, which ideally accounts for the difference in gene set sizes and correlations with phenotypes across gene sets.

#### 2.4.2 Over representation analysis

ORA was performed in the Database for Annotation, Visualization, and Integrated Discovery (DAVID) to determine gene ontology (GO) terms related to the dysregulated genes of CD, UC, and SI-NET identified by RRHO (Huang et al., 2009; Sherman et al., 2022). GO terms were subdivided into biological process (BP), cellular component (CC), and molecular function (MF) (Aleksander et al., 2023; Ashburner et al., 2000). Statistically significant GO terms were required to meet a Benjamini–Hochberg FDR <0.05 and were ranked based on statistical significance.

### 2.5 Network analysis

#### 2.5.1 Protein-protein interaction network construction

The protein-protein interaction network (PPI) of the co-DEGs between CD and SI-NET and UC and SI-NET was constructed through the Search Tool for the Retrieval of Interacting Genes (STRING) web-based platform version 12.0 (Szklarczyk et al., 2019). A medium confidence score of 0.400 was set. The PPI network was then transferred to the Cytoscape software version 3.10.1 for visualization (with Cytoscape yFile Layout Algorithm version 1.1.3.) and further topological analysis (Shannon et al., 2003).

#### 2.5.2 Sub-network and topological analysis of PPI

Molecular Complex Detection (MCODE) version 2.0.3, a Cytoscape add-on, was used to identify densely connected modules (Bader and Hogue, 2003). The parameters were as follows: degree cut-off = 2, node score cut-off = 0.2, K-core = 2, max. depth = 100. Additionally, modules with an MCODE score of less than five were discarded. Another Cytoscape plug-in, cytoHubba version 0.1, was used to identify hub genes (Chin et al., 2014). Considering the small scale of each PPI network, only the top five genes were identified based on the Maximal Clique Centrality (MCC) algorithm, which produced the most accurate results in a yeast PPI simulation out of the 11 topological analyses presented by cytoHubba.

#### 2.5.3 Enrichment analysis of protein interactions

The Cytoscape stringApp (version 2.1.1) group-wise functional enrichment tool was used to identify the biological functions of the individual and clustering proteins in the PPI network (Doncheva et al., 2019). String Enrichment employs an ORA-based method to identify significant biological pathways from 11 commonly used functional path classification frameworks; among which, we focused on GO terms and Reactome pathways to determine the biological relevance of the interaction networks and easier compare with other enrichment analysis results.

#### 2.5.4 Transcriptional and post-transcriptional network regulating hub genes

The correlation between IBD and SI-NET was finally analyzed at the transcriptional and post-transcriptional level through network analysis of transcription factors (TFs) and microRNAs (miRNA) regulating identified hub genes. JASPAR 9th edition is an open-access database of transcription factors for six taxonomic groups (Castro-Mondragon et al., 2022). DIANA-TarBase version eight is one of the largest micro-RNA (miRNA) interaction databases, encompassing roughly 670,000 miRNA target pairs (Karagkouni et al., 2018). JASPAR and DIANA-TarBase, along with NetworkAnalyst version 3.0 (Zhou et al., 2019), a web-based platform for topological analysis of gene expression data, was used to identify significant TF and miRNA associated with the top five hub genes in each of the four PPI networks. Cytoscape was used to merge, visualize, and identify critical TF and miRNA.

## 3 Results

### 3.1 Molecular signatures of CD, UC, and SI-NET

After data normalization and quality control, all active UC samples primary SI-NET samples, and their relative HC were selected for subsequent analyses. However, four samples (GSM1845783, GSM1945790, GSM1945798, and GSM1945832) from the CD dataset were identified as outliers with “*arrayQualityMetrics*”. These samples present with potential technological biases and therefore, are removed from further downstream analyses. With a statistical threshold of q-value FDR <0.01 and │log2(Fold Change) > 1.5│, a total of 1169 DEGs (675 upregulated and 494 downregulated) were identified between CD versus controls. Similarly, 2331 DEGs (1397 upregulated and 934 downregulated) and 4302 DEGs (2178 upregulated and 2124 downregulated) were identified in the UC and SI-NET cohorts respectively. The complete list of DEGs is documented in Supplementary Table S1.

**FIGURE 1:**
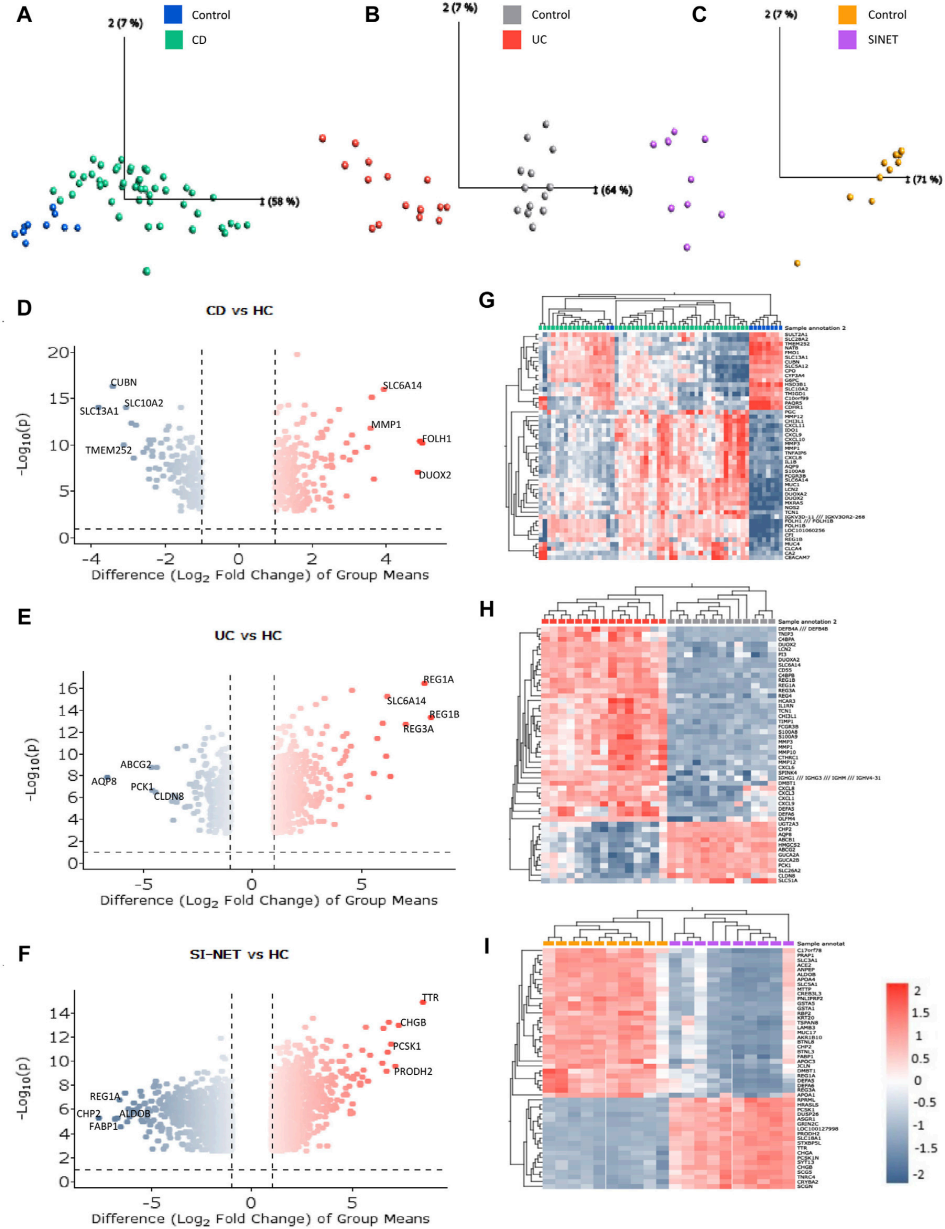
Molecular signatures of CD, UC, and SI-NET **(A–C)**: PCA plots comparing diseased and control samples. The PCA plots for CD, UC, and SI-NET respectively indicate clear separation across samples from all three diseases and their respective control groups, with a high percentage of variance explained by principal component 1. **(D–F)**: Volcano plots of differential expression analysis. The volcano plots depict the top four differentially expressed genes (DEGs) with both high fold change and statistical significance. **(G–I)**: Bidirectional hierarchical clustering heatmaps of the top 50 DEGs. The heatmaps show genes with the highest fold change and cluster samples based on similarity of expression levels. Two control samples are grouped among CD samples, indicating potential misclassification, biological variability, or a distinct subgroup of control samples that exhibit similar gene expression patterns to CD. These samples, nonetheless, are grouped among other controls when adjusting for higher q-value and fold change, eliminating the possibility of misclassification. The complete list of DEGs are listed in Supplementary Table S1.

Principal component analysis, an unsupervised dimensionality reduction method, indicated that diseased and healthy control tissues are more easily differentiated post differential expression analysis, with the principal component 1 effectively capturing 58%, 64%, and 71% of the total variance in the data of CD, UC, and SI-NET respectively (Figures 1A–C). Bidirectional hierarchical clustering (Figures 1G–I) also depicted a clear separation between expression levels of the top 50 DEGs in CD, UC, and SI-NET; still, two control samples displayed a similar expression profile to CD. These samples were still included for subsequent analysis, considering there is no evidence of technical errors and that the sample may provide insightful biological gene expression of normal ileum mucosa. This is because when adjusting for a higher q-value FDR and │log2(Fold Change)│, the samples were grouped among controls rather than CD. Figure 1 also displays the volcano plots of the top four DEGs with highest FDR and fold change in each disease state (Figures 1D–F), and Table 1 contains information on the selected dataset such as microarray platform, number of probe IDs, number of genes after conversion, and number of samples selected for analysis.

**TABLE 1:** Characteristics of the GEO datasets.

GSE	GPL	Microarray platform	Probes	Genes	Samples	Reference
75214	6244	Affymetrix Human Gene 1.0 ST Array	33297	20818	48 active CD & 10 HC	Vancamelbeke et al. (2017)
38713	570	Affymetrix Human Genome U133 Plus 2.0 Array	54675	22486	15 active UC & 13 HC	Planell et al. (2013)
65286	4133	Agilent-014850 Whole Human Genome 4 × 44K	45220	19750	10 primary SI-NET & 10 HC	Andersson et al. (2016)

### 3.2 Comparison of the molecular relationship between CD and SI-NET versus UC and SI-NET

**FIGURE 2:**
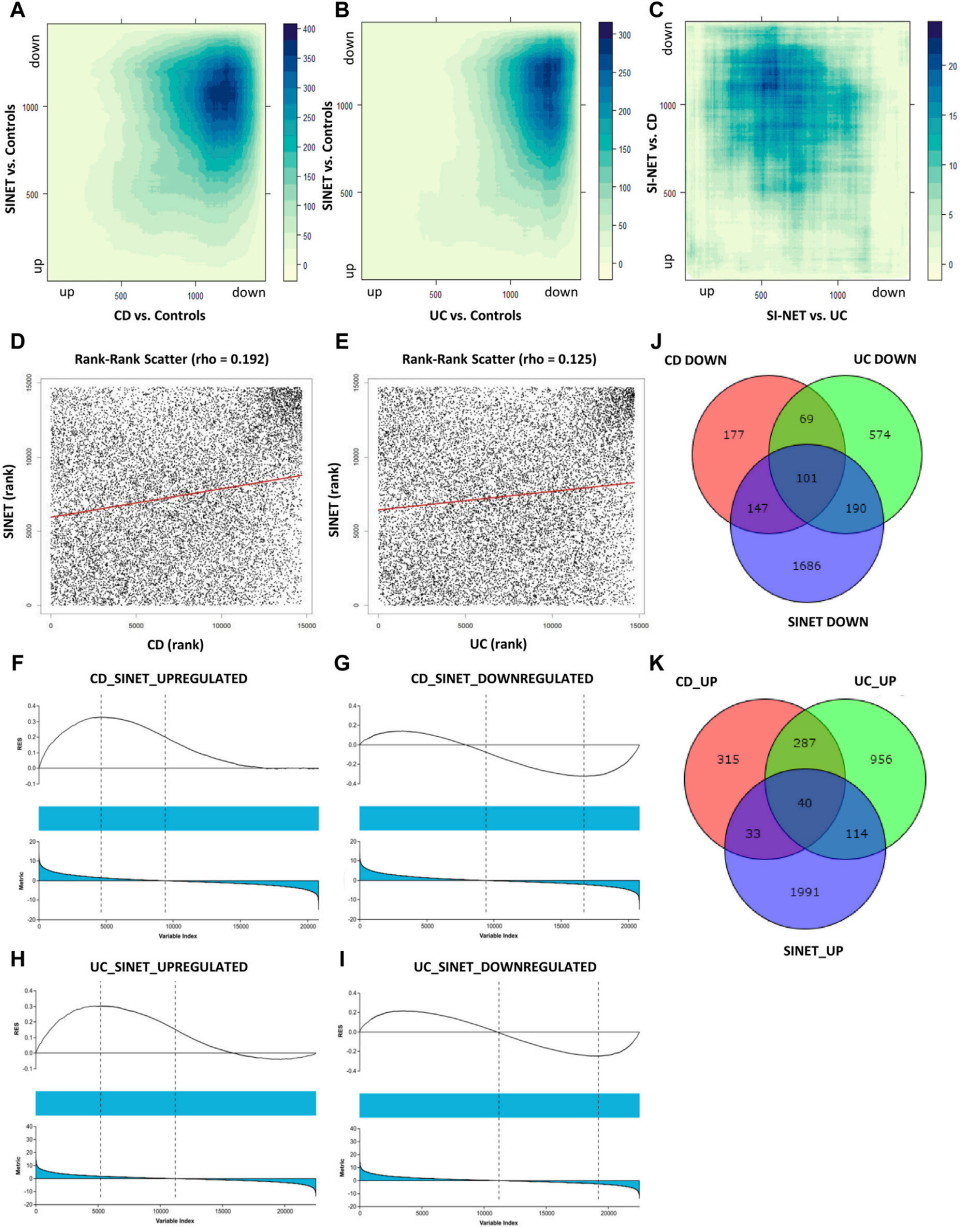
Comparison of gene expression between IBD Subtypes and SI-NET **(A–C)**: RRHO results comparing transcriptome-wide expression pattern between CD and SI-NET, UC and SI-NET, and the overlap of CD/SI-NET and overlap of UC/SI-NET shows highest overlap among downregulated genes (dark-blue colored). The accompanying color legend indicates the -log (*p*-value) of the region. Comparison of the overlap between CD/SI-NET and UC/SI-NET indicates that several clusters ranked at the top in UC and SI-NET are ranked at the bottom in CD and SI-NET, highlighting possible different biological mechanisms. **(D,E)**: scatter plot with the Spearman’s correlation coefficient (rho) of the overlap between CD and SI-NET and UC and SI-NET. Higher rho value between CD and SI-NET compared to UC and SI-NET suggests a closer molecular relationship between CD and SI-NET. **(F–I)**: GSEA validation of RRHO results, indicating higher similarity among downregulated genes between CD and SI-NET (NES = −1.46), whereas higher similarity among upregulated genes between UC and SI-NET (NES = 1.47). **(J,K)**: Venn diagram of overlapping differentially expressed genes between CD, UC, and SI-NET. These numbers are then used to compute the Jaccard similarity index, in which the index of UC and SI-NET (0.0719) is higher than of CD and SI-NET (0.0623). This shows that while CD and SI-NET might share a closer transcriptomic pattern, UC and SI-NET would share more exclusive dysregulated genes and biological pathways.

The ranked, unfiltered gene lists of each disease were compared with rank-rank hypergeometric overlap (RRHO) in RStudio. RRHO calculates the statistical significance of the coordinated changes in expression profiles of two gene sets with the hypergeometric distribution and the Spearman’s correlation coefficient. The most significant overlap in both UC and CD with SI-NET was seen in the dark blue-colored regions of the top-right quadrant of the RRHO heatmap (Figures 2A, B), which represents downregulated genes. However, UC downregulated genes, as indicated in the lighter blue regions, also have significantly more overlap with SI-NET upregulated genes than CD downregulated genes. Noteworthily, “*RRHOComparision*” results found several clusters of genes ranked at the top between UC and SI-NET while at the bottom between CD and SI-NET (Figure 2C), indicating potentially different molecular mechanisms. Overall, a stronger relationship between CD and SI-NET was found compared to UC and SI-NET, with a computed rank-rank scatter Spearman rho of 0.192 (Figure 2D) and 0.125 (Figure 2E), respectively. CD and SI-NET share a total of 4488 overlapping significant genes, while UC and SI-NET share 3236. Verification of RRHO results through gene set enrichment analysis (GSEA) Kolmogorov-Smirnov statistic indicated similar trends: between CD and SI-NET, a higher magnitude normalized enrichment score (NES) of −1.46 (FDR = 0.059829) was computed for matches (8217/10532) in downregulated genes, while the statistics are 1.38 (FDR = 0.090226) and 7910/10531 matches in upregulated genes (Figures 2F, G). Comparing the ranked gene lists of UC and SI-NET, a higher NES score of 1.47 (FDR = 0.029005) was seen among upregulated genes (8213/10531 matches) as compared to downregulated genes (NES = −1.14, FDR = 0.24477, and matches = 8413/10532), which accounts for the large overlap between downregulated UC DEGs with upregulated SI-NET DEGs (Figures 2H, I). Overall, both RRHO and GSEA suggest a moderate molecular relationship between both IBD phenotypes and SI-NET regarding gene expression levels.

In addition to comparing the whole transcriptomes of IBD and SI-NET, we also compare the lists of DEGs and compute the Jaccard Index (JI). Similar to RRHO results, there is a weaker correlation among upregulated genes (JI_CD_ = 0.0263 and JI_UC_ = 0.0450) than downregulated genes (JI_CD_ = 0.1046 and JI_UC_ = 0.1052). Though, a comparison of overlapping DEGs indicated that there is a stronger relationship between UC and SI-NET (overall JI_UC_ = 0.0719) as compared to CD and SI-NET (overall JI_CD_ = 0.0623), which is different from RRHO results. Among the shared dysregulated genes, 101 downregulated (23%) and 40 upregulated (21.4%) genes are found in both CD, UC, and SI-NET, indicating strong similarities between overlapping dysregulated genes of CD and SI-NET with that of UC and SI-NET. The discrepancies in JI analysis versus that of RRHO and GSEA imply that while there might be generally stronger similarities in transcriptome-wide gene expression profiles of CD and SI-NET, certain biological pathways might be more tightly linked between UC and SI-NET, considering a higher overlap of DEGs. The Venn diagrams of overlapping genes are displayed in Figures 2J, K while the complete lists of co-DEGs are documented in Supplementary Table S2.

Next, we compared the functional annotations related to the significant overlapping gene lists (identified by RRHO) of CD and SI-NET versus that of UC and SI-NET through over-representation analysis (ORA) of gene ontology (GO) terms, including biological processes (BP), cellular components (CC), and molecular functions (MF). A total of 127 GO terms (42 BP, 69 CC, and 16 MF) were enriched among the overlaps between upregulated genes of CD and SI-NET, whereas only 44 GO terms (11 BP, 26 CC, and 7 MF) were identified as statistically significant among downregulated genes (Supplementary Table S3). Between UC and SI-NET, 61 upregulated GO terms (8 BP, 40 CC, and 13 MF) and 28 downregulated GO terms (7 BP, 16 CC, and 5 MF) were found (Supplementary Table S4). We identified significant overlaps among the enriched functional annotations between GO terms of UC, CD, and SI-NET. Seven out of eight enriched GO processes are shared among all CD, UC, and SI-NET, most significantly “collagen fibril organization,” “cell adhesion,” and “extracellular matrix organization.” Among the top five enriched BP terms in CD and SI-NET upregulated genes, only “cilium assembly” was not found in UC. The term “microtubule cytoskeleton organization” is exclusively shared between UC and SI-NET. For GO components and GO functions, the top three CC terms in both overlapping lists are “nucleoplasm,” “cytosol,” and “cytoplasm” while the top 2 MF terms are “protein binding” and “extracellular matrix structural constituent.”

Similarly, the downregulated genes in either CD or UC that overlap with SI-NET partake in various metabolic processes, namely, “xenobiotic metabolic process,” “fatty acid metabolic process,” and “fatty acid beta-oxidation.” The main difference between shared biological pathways is that downregulated genes in CD and SI-NET also participate in various cholesterol and lipoprotein metabolic processes, whereas the rest of the genes shared between UC and SI-NET are more related to cellular energy metabolism and transport processes. The enriched GO components, however, differ: the top three enriched CC terms in CD are “apical plasma membrane,” “extracellular exosome,” and “brush border membrane,” whereas in UC, the “mitochondrion,” along with “mitochondrial matrix” and “mitochondrial inner membrane” play the most essential roles. Three shared GO functions are identified across all diseased states, including “RNA polymerase II transcription factory activity, ligand-activated sequence-specific DNA binding,” “PDZ domain binding,” and “flavin adenine dinucleotide binding.” Interestingly, the GO MF terms “protein binding” or “identical protein binding” were significantly represented in both sets of upregulated and downregulated genes across all disease states, highlighting the fundamental roles of protein interactions in the molecular pathology of CD, UC, and SI-NET.

The top three functional terms, along with the number of genes matches and Benjamini–Hochberg FDR, in each category (BP, CC, and MF) of the up-and downregulated genes shared between CD/SI-NET and UC/SI-NET are documented in Tables 2, 3 respectively.

**TABLE 2:** Top three enriched pathways among upregulated and downregulated overlapping genes between CD and SI-NET.

Regulation	Category	Term	Count	Benjamini
Upregulate	GO BP	Collagen fibril organization	33	5.90E-7
		Extracellular matrix organization	59	1.00E-6
		Angiogenesis	81	1.80E-6
Downregulate		Cholesterol metabolic process	24	5.80E-6
		Xenobiotic metabolic process	27	1.50E-5
		Lipid metabolic process	37	3.10E-4
Upregulate	GO CC	Cytoplasm	1128	8.30E-19
		Cytosol	1073	1.20E-12
		Nucleoplasm	798	6.20E-12
Downregulate		Apical plasma membrane	78	9.30E-21
		Extracellular exosome	221	3.00E-14
		Brush border membrane	25	3.90E-14
Upregulate	GO MF	Protein binding	2447	1.50E-29
		Extracellular matrix structural constituent	62	5.30E-15
		RNA binding	352	2.20E-9
Downregulate		Protein binding	917	1.30E-7
		RNA polymerase II transcription factor activity, ligand-activated sequence-specific DNA binding	18	1.70E-5
		Zinc ion binding	95	5.60E-4

**TABLE 3:** Top three enriched pathways among upregulated and downregulated overlapping genes between UC and SI-NET.

Regulation	Category	Term	Count	Benjamini
Upregulate	GO BP	Collagen fibril organization	23	1.50E-3
		Cell adhesion	101	1.50E-3
		Microtubule cytoskeleton organization	36	3.20E-3
Downregulate		Xenobiotic metabolic process	22	2.50E-6
		Fatty acid beta-oxidation	15	2.50E-6
		Tricarboxylic acid cycle	12	3.40E-5
Upregulate	GO CC	Nucleoplasm	571	3.10E-11
		Cytosol	743	1.60E-09
		Cytoplasm	754	1.60E-09
Downregulate		Mitochondrion	112	5.50E-13
		Mitochondrial matrix	42	3.30E-07
		Mitochondrial inner membrane	47	3.30E-07
Upregulate	GO MF	Protein binding	1716	9.80E-28
		Extracellular matrix structural constituent	42	3.50E-8
		RNA binding	136	5.90E-5
Downregulate		Identical protein binding	100	1.40E-2
		RNA polymerase II transcription factor activity, ligand-activated sequence-specific DNA binding	11	1.50E-2
		Transferase activity, transferring acyl groups	10	2.20E-2

### 3.3 Gene set enrichment analysis reveals two distinct biological pathways differentiating CD and SI-NET versus UC and SI-NET relationships

We performed Gene Set Enrichment Analysis (GSEA) on the complete ranked gene lists in each disease to identify relevant biological pathways in the Reactome repository. With the conventional statistical threshold of FDR <0.25, only three significantly enriched pathways were identified in the ranked gene list of SI-NET and two in CD. Recognizing the negative impact of small sample sizes on statistical power, we used a more lenient cutoff of NES > │1.5│ and FDR <0.45 to identify biologically relevant gene sets, yielding a broader set of enriched pathways: 90 in SI-NET (Supplementary Table S5), 67 in CD (Supplementary Table S6), and 199 in UC (Supplementary Table S7).

**FIGURE 3:**
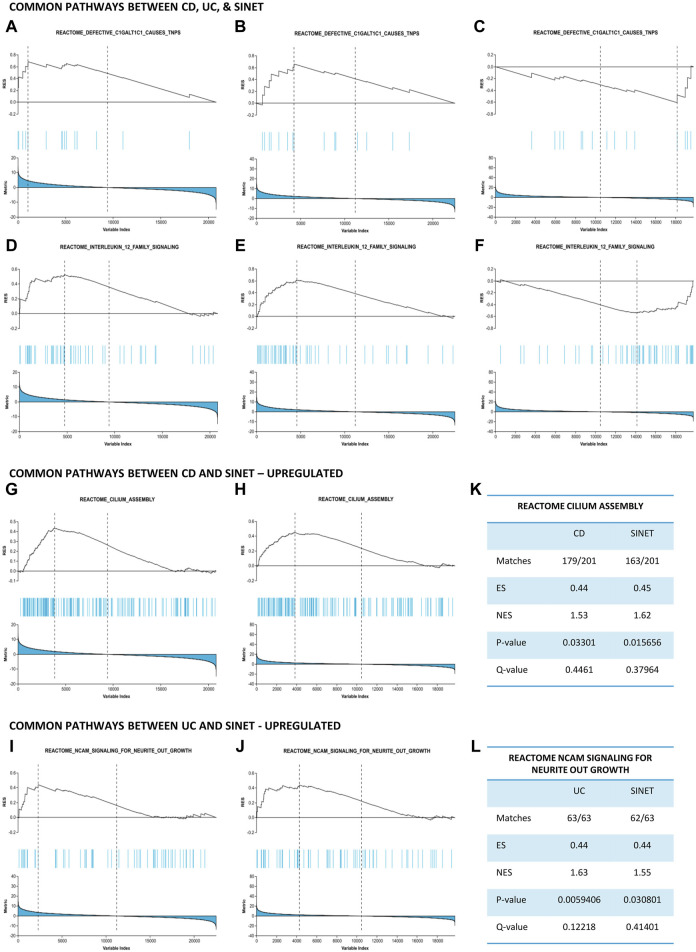
Gene set enrichment analysis identifies common pathways between IBD and SI-NET **(A–F)**: Pathways that are upregulated in IBD while downregulated in SI-NET, including “Defective C1GALT1C1 Causes TNPs” and “Interleukin 12 Family Signaling.” These pathways indicate different mechanisms related to C1GALT1C1-mediated glycoprotein synthesis and inflammatory signaling through Interleukin 12. **(G–J)**: Pathways that are common between SI-NET and each IBD subtypes. CD and SI-NET share the upregulation of “Cilium assembly,” implicating the role of ciliogenesis, particularly ciliary signaling, in the pathogenesis of these two diseases. UC and SI-NET share the upregulation of “NCAM Signaling Neurite Outgrowth,” which resembles a potential gut-brain axis in these two diseases. **(K,L)**: Statistical metrics related to the common pathways between IBD and SI-NET.

Thereafter, these pathways were compared to identify biological relationships between two IBD phenotypes and SI-NET at a systems biology level. Two pathways, namely, “Defective C1galt1c1 Causes Tnps” and “Interleukin 12 Family Signaling,” are strongly upregulated in CD and UC while downregulated in SI-NET (Figures 3A–F). Additionally, the regulation of several other pathways, including “Pyroptosis,” “Azathioprine Adme,” “Acyl Chain Remodeling Of Pg,” “Interleukin 12 Signaling,” and “Regulation Of Signaling By Cbl,” differs between UC and SI-NET. The contrasting regulation in these pathways indicates different biological mechanisms in IBD and SI-NET, particularly in several immune responses, cell death, and metabolic processes. Contrarily, we also identified two pathways with the same direction of regulation: “Cilium Assembly” (CD and SI-NET) and “Ncam Signaling for Neurite Outgrowth” (UC and SI-NET) are both significantly upregulated (Figures 3G–J). GSEA statistical metrics, including number of matches, enrichment scores, and false discovery rates, for these two pathways are documented in Figures 3K, L. The identification of “Cilum Assembly'' shared between CD and SI-NET through GSEA further corroborated with ORA results, which also identifies cilium assembly as the most significant pathway exclusively between CD and SI-NET. Thus, enrichment results suggest that ciliary function plays an indispensable role in the pathogenesis of both disease states, possibly through regulating cellular signaling and tissue integrity. In addition, UC and SI-NET shares the upregulation of NCAM signaling, which participates in neuronal structural developments and signaling, as well as cell adhesion. The enrichment of NCAM signaling and neuronal functions possibly reflects the neuroimmune interactions through the gut-brain axis in UC and neuroendocrine differentiation in SI-NET.

#### 3.3.1 Memory B Cells are core attributes of IBD and SI-NET immune responses while CD4^+^ helper T Cells, macrophages, and dendritic cells further characterize UC and SI-NET relationship

Given that the role of immune responses in SI-NET carcinogenesis is largely unexplored, we used GSEA to identify enriched immunological pathways in dysregulated SI-NET genes, focusing on common immune mechanisms with IBD. The “C7: immunologic signature gene set” includes gene expression observed across various immune cell types, states, and perturbations. Here, we identified 27 enriched gene sets for SI-NET, 486 for CD, and 1285 for UC with a statistical threshold of NES > │1.5│ and FDR <0.45. Interestingly, no statistically significant downregulated gene sets were identified, despite the prevailing notion of an immunosuppressive environment in SI-NET. Among the significant pathways, one of them was common across all conditions, while UC and SI-NET shared four additional pathways. The complete list of dysregulated immune pathways and relevant statistical metrics for SI-NET, CD, and UC are recorded in Supplementary Tables S5–S7 accordingly.

The shared upregulation of genes from “GSE11961: Plasma Cell Day 7 Vs. Memory B Cell Day 40 Downregulated” demonstrates a consistent immunological relevance across CD, UC, and SI-NET, with robust NES metrics of 1.94, 1.7, and 1.59 respectively. This gene set contains genes downregulated in plasma cells at day 7 but upregulated in Nitrophenyl-specific/IgG1-expressing memory B cells at day 40, indicating a strong connection to memory B cell development in all three diseases (Kaji et al., 2012). In addition to memory B cells, UC and SI-NET share similar molecular profiles in the regulation of macrophages, dendritic cells (DC), and CD4^+^ Th1. Specifically, the gene set “GSE19941: Unstimulated Vs. Lipopolysaccharide- & IL-10-Stimulated In *Nfkb1* ^*−/−*^ *Il10* ^*−/−*^ Macrophage Upregulated” (Yang et al., 2011) was particularly represented among upregulated genes in UC (NES = 1.52) and SI-NET (NES = 1.57). These genes are upregulated in macrophages lacking NFKB1 and IL10 compared to those stimulated with IL-10 and Lipopolysaccharide, which activates NFKB1 expression. This indicates a hyperactive inflammatory state driven by dysregulated NF-kB signaling in macrophages, particularly due to the loss of IL-10 suppression and the NFKB1 (p50) subunit. Similarly, gene expression patterns in UC and SI-NET suggest proactive immune responses in DC, where upregulated DEGs in UC and SI-NET correspond with upregulated genes in CpG DNA—an agonist of Toll-Like Receptor 9 (TLR9) that are recognized by the immune system as bacterial or viral DNA—stimulated DC compared to wild-types DC (GSE17721: Control 0 h Vs. CpG 0.5 h Dendritic Cells Downregulated) (Amit et al., 2009).

In both UC (NES = 1.56) and SI-NET (NES = 1.56), we observed an upregulation of genes associated with wild-type Th1 CD4^+^ cells compared to Erg2^−/−^ cells, as indicated by the gene set analysis from “GSE46242: Control vs. Erg2 Deleted Th1 CD4^+^ T Cells Upregulated” (Zheng et al., 2013). This upregulation corresponds to genes typically expressed in Th1 cells expressing Erg2, which are known to induce T cells’ anergy and contribute to peripheral tolerance. Despite the marked immunoreactivity seen in memory B cells, macrophages, and dendritic cells, the similar upregulation in Erg2-active Th1 cells may suggest a compensatory mechanism in UC aimed at reducing chronic inflammation. Additionally, this could reflect an adaptive strategy in the SI-NET microenvironment to evade immune surveillance through enhanced peripheral tolerance. Interestingly, comparing gene expression between macrophages and effector memory CD4^+^ cells using the ‘GSE3982: Macrophages vs. Effector Memory CD4^+^ T Cell Downregulated’ set shows that the upregulated genes in UC and SI-NET resemble the downregulated genes in macrophages (Jeffrey et al., 2006). This suggests stronger CD4^+^ immunosuppressive responses (peripheral tolerance) than proinflammatory responses mediated by macrophages with IFN-β.

### 3.4 Protein-protein interaction networks identifies central protein complexes in CD, UC, and SI-NET pathogenesis

Because enrichment analysis reveals that protein-protein interactions play an essential role in the disease pathogenesis of both IBD and SI-NET, we constructed protein interaction networks (PPI) among the overlapping DEGs as separated by direction of change. Hence, four interaction networks were generated with STRING-db and were inputted in Cytoscape for further subnetwork and topological analyses. Each node in the network represents a protein and each edge represents an interaction between two proteins. We thus filtered nodes that are not a part of the largest sub-network (that is, in our PPI networks, nodes that have no interactions or two nodes that have one, bidirectional interactions). Finally, we performed enrichment analysis to identify the biological function of the protein interactions in these densely connected submodules, and the complete list of functional annotations are listed in Supplementary Table S8.

**FIGURE 4:**
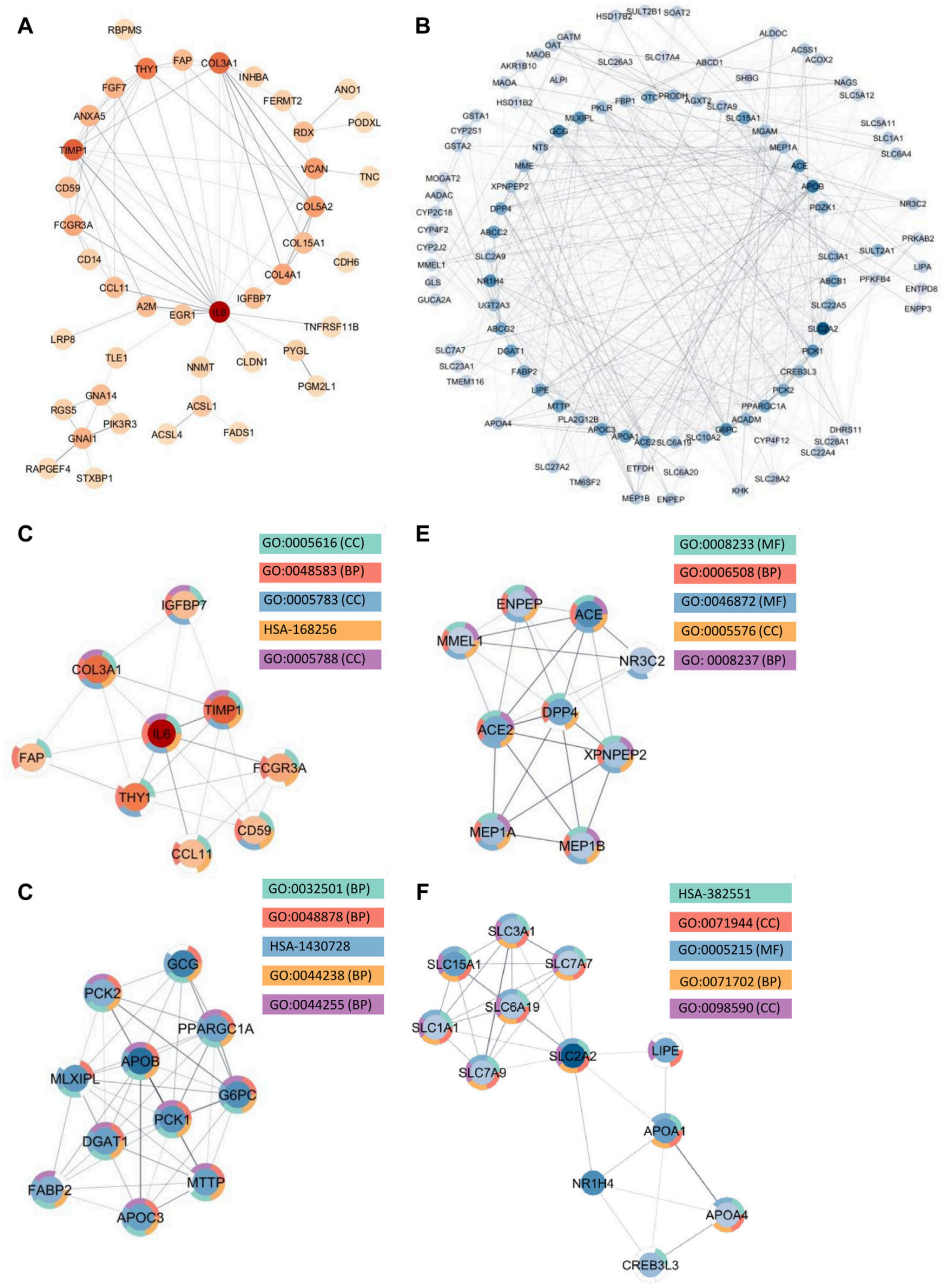
Protein Interactions and Network Analysis of co-DEGs Between CD and SI-NET **(A,B)**: Protein-protein interaction (PPI) network of upregulated (red color palette) and downregulated genes (blue color palette) shared between CD and SI-NET respectively. The darker tone indicates node (protein) with higher degree (interaction), and thicker edge indicates stronger relationship. The inner circle includes the most essential genes in the network. For visualization, the downregulated network only contains genes with at least 3°. **(C–F)**: Significant densely connected regions in the PPI network identified through the MCODE algorithm (red colors are upregulated modules and blue colors are downregulated modules). The darker nodes are the seed nodes (central-most important node) in the network. The color of the borderline resembles related significant enrichment terms. Overall, enrichment analysis indicates that the upregulated module mainly involves “Response to Stimulus” and “Immune System,” whereas the three other downregulated modules are related to various “Primary and Cellular Metabolic Processes.” While the upregulated genes in CD and SI-NET involve IL-6 mediated immune responses, many of the other genes are substantially related to the extracellular matrix (ECM), particularly fibroblast activation (FAP, COL3A1, TIMP1).

**FIGURE 5:**
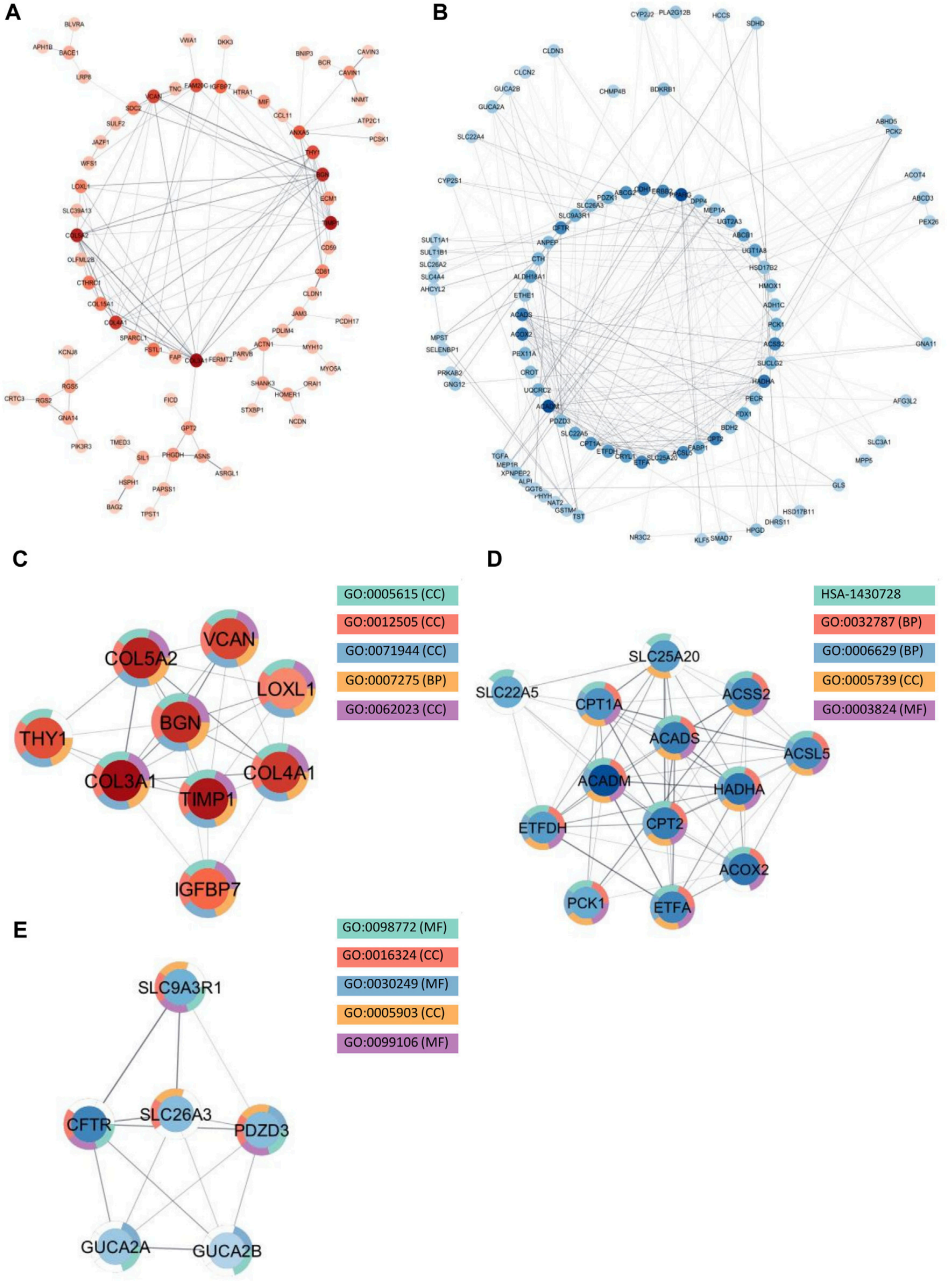
Protein interactions and network analysis of co-DEGs between UC and SI-NET **(A,B)**: Protein-protein interaction (PPI) network of upregulated (red color palette) and downregulated genes (blue color palette) shared between UC and SI-NET respectively. The darker tone indicates node (protein) with higher degree (interaction), and thicker edge indicates stronger relationship. The inner circle includes the most essential genes in the network. For visualization, the downregulated network only contains genes with at least 3°. **(C-E)**: Significant densely connected regions in the PPI network identified through the MCODE algorithm (red colors are upregulated modules and blue colors are downregulated modules). The darker nodes are the seed nodes (central-most important node) in the network. The color of the borderline resembles related significant enrichment terms. Overall, enrichment analysis indicates that the upregulated module mainly involves “Systems Development” and “Multicellular Organism Development” whereas the two other downregulated modules are related to “Fatty Acid and Lipid Metabolism.” Many of the upregulated genes are essential components in the extracellular matrix (ECM), indicating significant involvement of the ECM in UC and SI-NET pathogenesis.

Among the upregulated genes shared between CD and SI-NET, STRING-db generated a network containing 73 nodes and 82 edges (Figure 4A). Next, the MCODE algorithm was used to identify protein modules within the network (Figure 4C). MCODE identified one protein complex with a score of (5.25) that includes nine proteins and 21 interactions, among which, Interleukin-6 (IL-6) is the seed node. We then performed enrichment analysis to identify the molecular function of the identified protein complex, which participated in the process of “regulation of response to stimulus” and “immune system.” Similar to previous over-representation analysis, the gene products are mainly found in “extracellular space” and “endoplasmic reticulum.” The identification of this module as the most significant protein complex in the network and subsequent functional annotation unveil a shared upregulated immune mechanism behind CD and SI-NET that is particularly mediated by IL-6 in the extracellular space and the endoplasmic reticulum. In addition, we also found related molecular mechanisms amongst UC and SI-NET PPI of upregulated genes. The PPI contains 154 proteins and 144 interactions (Figure 5A), and sub-network analysis also found one significant module with an MCODE score of 7 (9 nodes and 28 edges) (Figure 5C). This subnetwork and that of CD and SI-NET both contain COL3A1, IGFBP7, THY1, and TIMP1, proteins that are crucial for extracellular matrix organization, tissue repair, and cell adhesion. These proteins might be particularly essential in cilium assembly and neuronal structural developments, which are significant shared pathways identified with GSEA. Enriched GO BP terms also supported such a notion, indicating that the protein complex notion, indicating that the protein complex shared between UC and SI-NET is heavily involved in “multicellular organism development” and “system development.” Thus, the main difference between the upregulated protein networks of CD and SI-NET vs. UC and SI-NET is the larger involvement of IL-6-dependent immune responses presented in CD and SI-NET.

We identified much larger networks among downregulated PPI, which reflects the larger overlap among downregulated genes identified with RRHO and GSEA. Specifically, the network constructed based on shared downregulated genes of CD and SI-NET involves 246 nodes and 569 edges (Figure 4B). Subsequently, three protein complexes with strongly related molecular functions were identified with MCODE. Cluster 1 (Figure 4D) (MCODE = 9) includes 11 proteins and 45 interactions, and is most significantly involved in “metabolism” (i.e., “primary metabolic process” and “cellular metabolic process”), “chemical homeostasis,” and “multicellular organismal process.” Cluster 2 (Figure 4E) (MCODE = 6.25, nodes = 9, edges = 25) is more involved in enzymatic activities that regulate protein catabolism and formation, such as “peptidase activity” or “proteolysis”. Moreover, analysis at the molecular level indicates that these proteins partake in the bindings of metal and zinc ions, which play essential roles in the formation and structural stabilization of the peptidase enzyme family. The last Cluster 3 (Figure 4F) (MCODE = 5.636, nodes = 12, edges = 31) located in the PPI network of CD and SI-NET downregulated genes includes proteins related to “transmembrane transporter activities” in the “cell periphery”, primarily of “small molecules” and “organic substances”. The molecular processes in Cluster three are critical for nutrient importation and waste product exportation, which are fundamental to metabolism. Similar to CD, the PPI network (290 proteins, 480 interaction) (Figure 5B) of overlapping downregulated genes of UC and SI-NET are heavily involved in metabolism with two Clusters found. However, instead of protein metabolisms, Cluster 1 (Figure 5D) of UC (MCODE = 10.167, nodes = 13, edges = 61) participates more in the metabolism of monocarboxylic, lipid, and fatty acid. Moreover, these proteins are mainly found in the mitochondrion and are molecularly involved in catalytic activity. The other module, Cluster 2 (MCODE = 5.2, nodes = 6, edges = 13) (Figure 5E), plays a broader role in regulating molecular functions, such as the ion channel or the guanylate cyclase. To conclude, analyzing the protein interaction mechanisms of downregulated genes reveals different metabolic processes: while CD and SI-NET downregulated genes are more involved in protein catabolism, UC and SI-NET downregulated genes regulate the process of lipid metabolism and catalyst activities.

#### 3.4.1 Molecular functions and regulatory network of central proteins in CD and SI-NET

**FIGURE 6:**
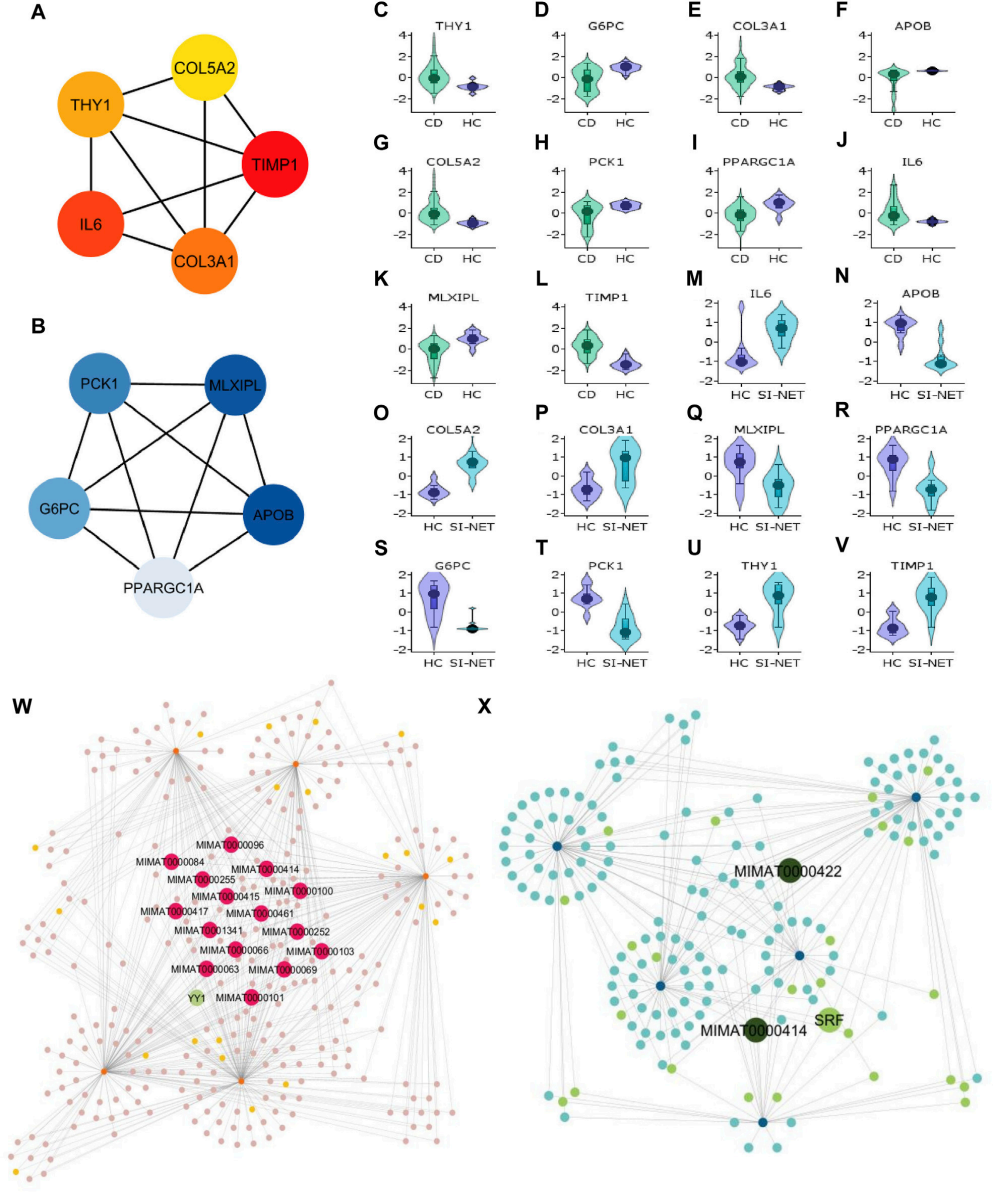
Analysis of hub genes and construction of regulatory networks between CD and SI-NET **(A,B)**: The top five most important genes in the networks of upregulated (red color) and downregulated (blue color) common DEGs between CD and SI-NET. Most of these genes are presented in the protein clusters identified through MCODE and suggest similar biological mechanisms; however, there are more presence of extracellular matrix-related proteins compared to immune-related proteins among the upregulated network. This indicates strong immune-ECM interactions in the disease states of CD and SI-NET. **(C–V)**: Expression levels of the 10 hub genes in CD and SI-NET through volcano plots, which indicates clear distinction in expression levels for most genes. **(W,X)**: Construction of the regulatory networks, including transcription factors (TF) and microRNA (miRNA) regulating the hub genes. 15 miRNAs are identified to regulate all five upregulated hub genes while only two miRNAs are known to regulate with all five downregulated hub genes. Among them, MIMAT0000414 interacts with all 10 hub genes, indicating dual regulation. The TF with the highest interaction is YY1 (4 interactions) among upregulated hub genes and SRF (3 interactions) among downregulated hub genes.

Aside from evaluating the biological properties of important protein complexes, we also found significant proteins (i.e., hub genes) that have a large impact on the PPI network. These proteins would display strong interactions with many other proteins in the network, which are usually reflected through the degree and strength of interaction. Here, we used the MCC algorithm to identify the top five most important nodes in the four PPI networks. Among the upregulated nodes shared between CD and SI-NET, four of the five hub genes are presented in the one protein complex identified, with COL5A2 as the only exception (Figure 6A). The other four genes include COL3A1, IL6, THY1, and TIMP1. Henceforth, the biological functions of these hub genes would be closely similar to the description of protein modules, underscoring the dynamic interaction between immune responses (IL6), collagen-fibril organization in the extracellular matrix (TIMP1, COL3A1, and COL5A2), and cell-cell or cell-extracellular matrix interactions (THY1). Unsurprisingly, the five central downregulated hub genes (APOB, G6PC, MLXIPL, PCK1, and PPARGC1A) are all seen in Cluster 1 (the central-most module in the network) of CD and SI-NET downregulated PPI (Figure 6B). Interestingly, these downregulated hub genes share a closer biological function with the modules identified by downregulated genes shared between UC and SI-NET, such as lipid metabolism.

We assessed the expression levels of these central genes among diseased and control cohorts with Violin plots to further validate their significance before analyzing their regulatory networks (Figures 6C–V). Thereafter, we constructed a hub gene-regulatory network with NetworkAnalyst to identify transcription factors (TF) and micro- RNA (miRNA) that associate with the hub genes. Among the upregulated hub genes, 301 miRNA and TF have 567 interactions with the five hub genes (Figure 6X), and among them, 15 miRNAs interact with all five hub genes and 1 TF (YY1) has strong connections with four of the five hub genes (all but COL5A2). Surprisingly, we only discovered two miRNA (MIMAT0000422 and MIMAT0000414) that engage with all downregulated hub genes and 1 TF (SRF) connecting with three hub genes (APOB, MLXIPL, and PCK1) in a network of 168 miRNA and TF that have 258 interactions with the hub genes (Figure 6X). MIMAT000414, however, is also identified among the 15 miRNAs that regulate all five upregulated hub genes, suggesting dual regulation and significant modulatory effects on the biological pathways shared between CD and SI-NET.

#### 3.4.2 Molecular functions and regulatory network of central proteins in UC and SI-NET

**FIGURE 7:**
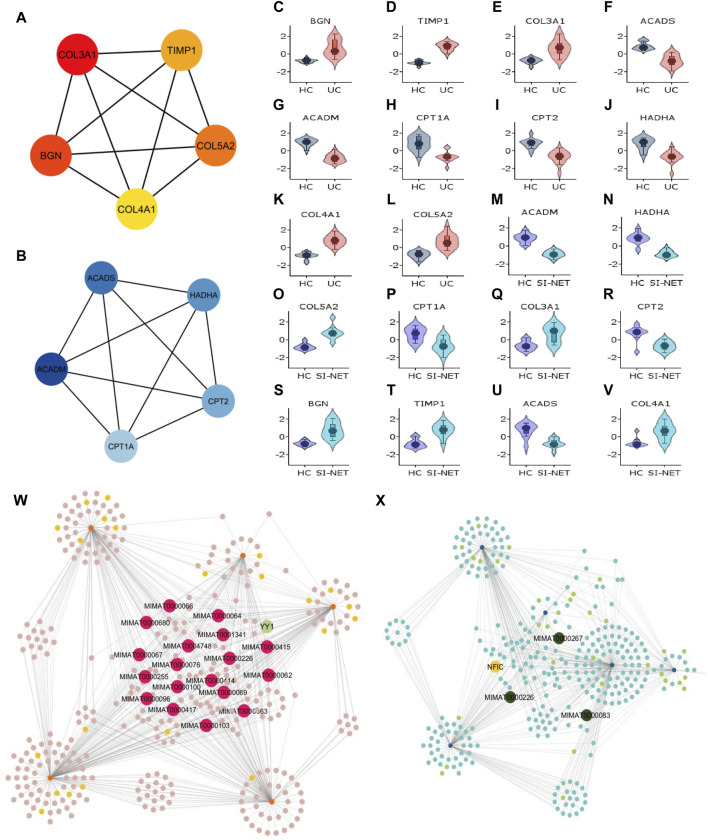
Analysis of hub genes and construction of regulatory networks between UC and SI-NET **(A,B)**: The top five most important genes in the networks of upregulated (red color) and downregulated (blue color) common DEGs between UC and SI-NET. Most of these genes are presented in the protein clusters identified through MCODE and suggest similar biological mechanisms; however, there is a significant presence of extracellular matrix-related proteins (TIMP1, BGN), particularly in collagen synthesis (COL3A1, COL4A1, COL5A2). This indicates that UC and SI-NET pathogenesis involves significant ECM accumulation, and possibly fibrosis. **(C–V)**: Expression levels of the 10 hub genes in UC and SI-NET through volcano plots, which indicates clear distinction in expression levels for most genes. **(W,X)**: Construction of the regulatory networks, including transcription factors (TF) and microRNA (miRNA) regulating the hub genes. 18 miRNAs are identified to regulate all five upregulated hub genes while only three miRNAs are known to regulate with all five downregulated hub genes. The TF with the highest interaction is YY1 (3 interactions) among upregulated hub genes and NFIC (4 interactions) among downregulated hub genes.

Recognized upregulated (BGN, COL3A1, COL4A1, COL5A2, and TIMP1) (Figure 7A) and downregulated (ACADS, ACADM, CPT1A, CPT2, and HADHA) (Figure 7B) hub genes by MCC in UC and SI-NET PPI network are all observed in previously identified MCODE clusters (Cluster 1 of each group). Noteworthily, three of the five upregulated hub genes in UC, namely, COL3A1, COL5A2, and TIMP1, are also identified among the PPI network of CD and SI-NET. These genes represent the most significant shared molecular mechanisms between CD, UC, and SI-NET, which is the involvement of tissue remodeling and cell adhesion. The main difference, nonetheless, involves the presence of immune proteins like IL-6 in the upregulated hub genes and modules of CD, indicating possible involvements of immune responses exclusively shared between CD and SI-NET. Among the downregulated hub genes, we did not identify any overlapping genes.

Similar to CD and SI-NET hub genes, assessments of UC and SI-NET hub genes as candidate markers for each disease were determined with Violin plots of their expression levels (Figures 7C–V), which indicates a clear difference between diseased versus control groups. The construction of regulatory networks by NetworkAnalyst identified 345 TFs and miRNAs sharing 650 connections with the upregulated hub genes (Figure 7W) and 325 TFs and miRNAs having 554 interactions with the downregulated hub genes (Figure 7X). We found 18 miRNAs that regulate all five upregulated hub genes and 1 TF (YY1) that regulate three of the five hub genes (COL3A1, COL4A1, and TIMP1). Among the downregulated hub genes, three miRNAs, including MIMAT0000983, MIMAT0000226, and MIMAT0000267, interacts with all hub genes, and 1 TF (NFIC) regulates all but ACADS. Noteworthily, the miRNA MIMAT0000226 is related to all ten hub genes (both up- and down-regulated) of UC and SI-NET, suggesting a dual role in the regulatory networks and the disease pathogenesis of UC and SI-NET.

Remarkably, we identified YY1 as having the most interactions among all TFs in both upregulated regulatory networks of CD/UC and SI-NET. This TF plays an essential role in regulating collagen fibrosis-related genes (COL3A1, COL4A1, TIMP1), immune-related gene (IL6), and cell-extracellular matrix interaction regulatory gene (THY1), which are all key pathways shared between IBD and SI-NET demonstrated by enrichment analysis. Moreover, 11 miRNAs are shared between the two upregulated regulatory networks. Thus, YY1, alongside with the 11 shared miRNAs between CD, UC, and SI-NET regulated networks, possibly play essential roles as candidate markers in both IBD and SI-NET.

## 4 Discussion

To the best of our knowledge, we report for the first time documenting the molecular relationships between IBD, one of the most prevalent diseases worldwide (Wang et al., 2023), and SI-NET, the most common malignancies of the small intestine (Barsouk et al., 2019). We compared the molecular profiles between two IBD subtypes, CD and UC, and discovered that while CD and SI-NET share a more similar global transcriptomics pattern, UC and SI-NET have a higher proportion of overlapping DEGs, suggesting possible dysregulated biological pathways exclusive for UC and SI-NET. We identified a total of 321 co-DEGs (248 downregulated and 73 upregulated) between CD and SI-NET and 445 co-DEGs (291 downregulated and 154 upregulated) between UC and SI-NET, with an overall Jaccard similarity index of 0.0623 and 0.0719 respectively. Our analysis indicates that UC and SI-NET molecular profiles are significantly similar, considering that the Jaccard similarity index between IBD and colorectal cancer, the most common IBD-associated malignancy, is only 0.076 (Al-Mustanjid et al., 2020). At the systems biology level, we identified gene ontology (GO) annotations related to collagen fibril organization, cell adhesion, and extracellular matrix organization that are significantly expressed among upregulated genes shared between SI-NET and both IBD phenotypes, whereas the downregulated genes participate in different metabolic processes of lipid, fatty acid, and glucose. We also found two pathways shared with SI-NET that are specific to each IBD subtype, namely, the upregulation of cilium assembly with CD and NCAM signaling with UC. Immunologically, pathways related to memory B cell formation are significantly upregulated in IBD and SI-NET, whereas UC and SI-NET share exclusive involvements of NF-kB dysregulated macrophages, pro-inflammatory TLR-9 expressing dendritic cells, and anergic CD4^+^ T cells. Finally, through network analysis, we reported 7 protein complexes, 17 most central hub genes, 11 miRNA, and 1 TF as candidate markers of both IBD and SI-NET.

Earlier studies have documented the complex, bidirectional interplay between the extracellular matrix (ECM), chronic inflammatory responses, and cancer growth [reviewed by Marangio et al. (2022)]. In short, unresolved chronic inflammation, as presented in IBD, induces production of ECM-modifying enzymes that results in ECM degradation, which is characterized by epithelial-mesenchymal transition, genome instability, metabolism reprogramming, and immune evasion (Landskron et al., 2014). Such remodeling of the ECM has been shown to promote carcinogenesis in SI-NET, with a particular emphasis on the overproduction of collagen III fibers, myofibroblasts, and profibrotic growth factors (e.g., serotonin, transforming growth factor beta (TFG-β), and PDGF) [reviewed by Cives et al. (2019)]. In our study, we identified that dysregulation of the ECM is consistently enriched with GSEA and ORA analysis of statistically significant shared genes from RRHO analysis and key protein complexes and hub genes from the PPI network of co-DEGs. Noteworthily, our network analysis pinpointed TIMP1 as the main tissue inhibitor of matrix metalloproteinase underlying ECM dysregulation, as it consistently appears as one of the central-most important genes in PPI networks of upregulated genes shared by SI-NET and both IBD subtypes. Previous analysis of TIMP1 expression levels using immunohistochemistry has shown significantly elevated levels in gastroenteropancreatic (Voland et al., 2008), bronchopulmonary (Blicharz-Dorniak et al., 2012), and primary skin (Massi et al., 2003) neuroendocrine tumors as well as the glandular epithelium of IBD (Jakubowska et al., 2016). However, our study is the first to pinpoint the biological significance of TIMP1 as the main regulator of ECM remodeling in SI-NET through bioinformatics analysis. Specifically, TIMP1 inhibits the function of matrix metalloproteinase (MMP), key regulators in ECM degradation, and an imbalance of TIMP1/MMP expression in which TIMP1 is upregulated results in enhanced proteolysis and eventually ECM accumulation or fibrosis (Arpino et al., 2015). Besides TIMP1, our network analysis identified other prominent upregulated genes involved in ECM structural components (COL3A1 and COL5A2), cell adhesion (THY1), and growth factor binding (IGFBP7). Our findings of increased collagen fibril-associated gene expression in this study align with previous research demonstrating elevated collagen type III synthesis in both IBD and SI-NET, reflecting the state of ECM accumulation (Cives et al., 2019; Domislovic et al., 2022).

One of the key characteristics of ECM accumulation is the activation of myofibroblasts, which can be produced through the epithelial-mesenchymal transition or fibroblast-to-myofibroblast transition. ORA analysis in our study indicates that the shared upregulated genes in IBD and SI-NET are enriched in the transition from epithelial cells to mesenchymal cells, indicating that this process is the main pathway for myofibroblast activation. In SI-NET particularly, the functional interaction between mesenchymal cells and NET cells is essential for tumor cell proliferation, which can only be regulated by ɑ-smooth muscle actin-positive (ɑ-SMA) myofibroblast [reviewed by Cives et al. (2019)]. The same review denotes that ɑ-SMA myofibroblast promotes NET cell proliferation through secreting IL-6, VEGF, and monocyte chemoattractant protein 1, and this protumorigenic effect is exclusive through paracrine signaling. In our study, we identified IGFBP7 as one of the most important upregulated genes through module analysis. Interestingly, previous studies of IGFBP7’s role in colorectal cancer have documented its potential function as a paracrine signaling molecule in the tumor-stroma crosstalk (Rupp et al., 2015). Moreover, our analysis revealed significant related GO terms among upregulated genes, namely, “actin cytoskeleton,” “actin binding,” and “actin filaments,” that are crucial for enabling α-SMA myofibroblasts contraction and exertion of mechanical forces on the ECM (Shinde et al., 2017). Interestingly, other genes in the protein module like THY1 and TIMP1 also indicate the transition from fibroblast to myofibroblast process, which is activated by mesenchymal cells, to partake in myofibroblast activation. Fibroblast-to-myofibroblast differentiation, as assessed by the expression of ɑ-SMA protein, was observed only among myometrial and orbital fibroblasts expressing THY1 (Koumas et al., 2003). Additionally, in a study on urethral scar tissues, TIMP1 expression strongly correlates with increased expression of fibroblast cell growth and migration that is dependent on the ERK/MAPK signaling pathways, as well as fibroblast-to-myofibroblast transition key factors, including ɑ-SMA (Sa et al., 2015). To summarize, we identified that TIMP1-induced ECM accumulation, as characterized by increased levels of collagen III and the presence of ɑ-SMA myofibroblasts, is the key biological pathway underlying IBD and SI-NET pathogenesis.

Our network analysis and GSEA results, however, suggest different immune mechanisms regulating ECM-related pathways shared between SI-NET and IBD that are exclusive to each IBD subtype of CD and UC. In a PPI network of overlapping CD and SI-NET upregulated genes, we identified IL-6 as the seed node in the most densely connected protein complex that relates to ECM remodeling. Previously, it has been known that CD expresses a higher level of IL-6 as compared to UC and IL-6 plays a fundamental role in sustaining chronic inflammation through the upregulation of anti-apoptosis proteins in CD4^+^ T cells (Alhendi and Naser, 2023). Moreover, IL-6, alongside TFN-ɑ, is currently the only two known cytokines that express higher levels in gastroenteropancreatic neoplasms (Mahečić et al., 2020). IL-6, as aforementioned, is secreted by ɑ-SMA myofibroblast upon activation. In return, it promotes the progression of intestinal fibrosis by inducing the proliferation of resident fibroblast and production of PDGF, as well as regulating mRNA and protein levels of TGF-β, TFG-β RII, and STAT-3 (Speca et al., 2012). Of particular interest, IL-6 expression levels are remarkably higher in CD samples with mesenchymal cells (Ito, 2003), and its neutralization improved fibrosis in chronic cardiac allografts (Diaz et al., 2009). Thus, we identified IL-6 as the immune mediator of ECM accumulation specifically between CD and SI-NET. While we did not detect any inflammatory protein among upregulated genes of UC and SI-NET, our GSEA results indicate the shared upregulation of various immune cells, including macrophages, dendritic cells, and anergic CD4^+^ T cells. We believe that NF-kB expressing macrophages, linked to NFKB1 downregulation and associated with heightened inflammatory responses in macrophages (Somma et al., 2021), would have the most significant effect on intestinal fibrosis in UC and SI-NET. This is achieved through stimulation of fibroblast to produce ECM components, promotion of epithelial-to-mesenchymal transition, and induction of macrophage polarization toward a pro-fibrotic M2 subtype (Kondaiah, 2023; Wang et al., 2024). However, considering that most of the above influences of NF-kB are identified in pulmonary fibrosis, further studies are required to validate its impacts on IBD and SI-NET-related intestinal fibrosis. We found extremely few relationships between TLR9-expressing dendritic cells and anergic T cells in fibrosis progression. To conclude, analyses of PPI and GSEA suggest the presence of cytokine IL-6 as the main immune driver of ECM accumulation in CD and SI-NET, whereas UC and SI-NET involves a more complex network of interactions with macrophages, dendritic cells, and anergic T cells–an area that requires further research.

Second to different immunological regulatory processes, certain signaling pathways involved in IBD and SI-NET intestinal fibrosis and fibroblast-to-myofibroblast transition are specific among IBD subtypes. GSEA results suggest that ECM accumulation manifested in CD/SI-NET and UC/SI-NET might have a bidirectional influence on ciliogenesis and NCAM signaling for neurite developments, respectively. The specific role of cilia in inflammation and carcinogenesis remains largely controversial across different studies: for instance, losses of primary cilia are linked with worsen inflammation and carcinogenesis (Paul et al., 2022), while its presence and development also serve as potential tumor markers, such as for pituitary neuroendocrine tumors (Martínez-Hernández et al., 2024). Regarding the specific context of fibrosis, nonetheless, it is observed that the presence of primary cilium and ciliogenesis are indispensable for pulmonary and cardiac fibrosis through regulation of the Sonic Hedgehog, TGF-β, or SMAD3 signaling pathway (Lee et al., 2018; Villalobos et al., 2019). These pathways, interestingly, can induce ciliogenesis in return. Similarly, in UC and SI-NET, NCAM (neural cell adhesion molecule) signaling and its relation to intestinal fibrosis present one of the potential gut-brain axis involved in disease mechanisms. NCAM-stimulated neuronal differentiation through protein kinase C and MAPK activation are heavily dependent on its interaction with the fibroblast growth factor receptor (FGFR). Although the role of NCAM in intestinal fibrosis is not as clearly defined, studies have shown that in renal fibrosis, the interaction between NCAM and FGFR1, as induced by TGF-β1, is crucial for facilitating the epithelial-to-mesenchymal transition (Životić et al., 2018). Overall, these findings suggest that the intestinal fibrosis of IBD and SI-NET involves a complex signaling network that is specific among IBD subtypes.

One of the most significant consequences of intestinal fibrosis is metabolic reprogramming, for it alters the normal intestinal tissue architecture, impairing nutrition absorptions, affecting gut motility, disrupting the intestinal microbiota, and introducing hypoxia, all of which contributes to malabsorption (Zhan et al., 2021). It has been recorded that fibroblasts that are involved in intestinal fibrogenesis show limited function in the metabolism of fatty acids while demanding more glucose and glutamine [reviewed by Bos and Laukens (2020)]. Our study documents similar dysregulation: we identified that genes and GO terms, such as “fatty acid β-oxidation,” “fatty acid catabolic process,” “mitochondrial fatty acid β-oxidation of saturated fatty acids,” and “fatty acid β-oxidation using acyl-CoAdehydrogenase,” are consistently downregulated. Specifically, all five overlapping downregulated genes (ACADM, ACADS, CPT1A, CPT2, HADHA) of UC and SI-NET are involved in various aspects of fatty acid metabolism, particularly mitochondrial fatty acid β-oxidation. Among the downregulated hub genes of CD and SI-NET, we also found the dysregulation of lipid metabolism (APOB), oxidative phosphorylation (PPARGC1A), and glycogenolysis and gluconeogenesis (G6PC, MLXIPL, and PCK1). Typically, myofibroblast activation and ECM accumulation demand high energy resources, suggesting an expected upregulation of lipid metabolism as a primary energy source for ATP generation. Nonetheless, our study and many others suggest a distinct downregulation of lipid metabolism in intestinal fibrosis, particularly in expression levels of related genes and metabolites (Wu et al., 2023). This dysregulation is believed to be mediated by TGF-β, which downregulates PPARs transcription factors essential for fatty acid uptake and oxidation. The downregulation of PPARs further exacerbates intestinal fibrosis, for PAAR inhibits ECM transcription and promotes internalization and degradation [reviewed by Bos and Laukens (2020)].

The dysfunction of lipid metabolism, henceforth, results in additional needs for the metabolism of glucose, the other source of cellular energy. In the context of SI-NET and IBD, such metabolism was mainly performed through glycolysis as per the Warburg effect, a phenomenon in which cancer cells prefer glycolysis over oxidative phosphorylation (regulated by PPARGC1A) to produce glucose, even in the context of ample oxygen (Liberti and Locasale, 2016). The same study explains that this phenomenon arises since glycolysis, while not as efficient as mitochondrial oxidative phosphorylation, produces energy at a faster rate that is readily available for cancer cells. However, enrichment analysis in our paper also suggests another mechanism: our study indicates impaired function of the mitochondria, which is essential for oxidative phosphorylation. Additionally, the activation of aerobic glycolysis also requires the dysregulation of glycogenolysis, whose enzymes-PEPCK, FBPase, and G6Pase-antagonize glycolysis function, particularly in the cancer context (Wang and Dong, 2019).Our study found that G6PC is notably downregulated, suggesting its key role as a suppressor of aerobic glycolysis in IBD and SI-NET. Similarly, gluconeogenesis is counterproductive to glycolysis as it reverses seven of the ten main reactions involved in glycolysis, except for pyruvate to PEP conversion, fructose 1,6-bisphosphate to fructose 6-phosphate conversion, and glucose 6-phosphate to glucose conversion (Gupta and Gupta, 2021). The distinct downregulation of PCK1 and of proteolysis, which feeds amino acid substrate into the gluconeogenesis cycle, in our study suggests gluconeogenesis malfunction as to promote the glycolytic switch. This metabolic reprogramming toward a glycolytic switch, while induced by intestinal fibrosis, also partakes to further exacerbate fibrosis by providing ATP to suffice the high energy demand for myofibroblast activation [reviewed by Bos and Laukens (2020)].

We further decipher the regulatory networks of these central genes and biological pathways by constructing networks of TF and miRNA that regulate the hub genes. For this, we rank each node based on the degree of interactions, with TFs and miRNAs that regulate more genes considered as more significant. Thus, we found a total of 11 miRNAs that regulate all ten upregulated genes of both IBD subtypes and SI-NET, and 1 TF (YY1) that regulate seven of the ten upregulated hub genes. We found no similar miRNA or TF in the regulatory network of downregulated hub genes. Among the 11 miRNAs, four are members of the let-7 miRNA (miR-7) family, including hsa-let-7b-5p (MIMAT0000063), hsa-let-7e-5p (MIMAT0000066), hsa-let-7g-5p (MIMAT0000414), and hsa-let-7i-5p (MIMAT0000415). The functionality of miR-7 has been implicated in the pathogenesis of various autoimmune diseases, including IBD; however, results are inconsistent. Specifically, it is described that miR-7 upregulation mitigates intestinal inflammation in IBD by inhibiting the RNF183 protein, which induces NF-kB activation (Yu et al., 2016). In contrast, miR-7 upregulation has also been documented in IBD-diseased tissues and intestinal epithelial cells (IEC), promoting IEC proliferation and inflammatory cytokines secretion (Chen et al., 2023). This indicates that deficiency of miR-7 can ameliorate the pathological damages of IBD. As for most malignancies, miR-7 acts as a tumor suppressor by disrupting the PI3K/Akt signaling pathway for cellular proliferation and migration, and its downregulation is also related to decreased effectiveness of cancer therapies (Gajda et al., 2021; Chen et al., 2023). Still, several other studies analyzing miR-7-5p level in SI-NET indicate strong upregulation in both primary and liver metastasis tumor samples as compared to healthy controls (Malczewska et al., 2018). The specific biological impact of this upregulation, however, remains unexplored. Interestingly, miR-7 upregulation, through targeting various signaling pathways like EGFR or FAK, is linked to the reversal of the transition from epithelial to mesenchymal cells in cancer of the liver, ovary, or breast (Kong et al., 2012; Zhou et al., 2014; Zhang et al., 2020). This indicates a more nuanced role of miR-7 in the pathogenesis of IBD and SI-NET, particularly in the context of intestinal fibrosis, that is yet to be uncovered. Aside from the miR-7 family, only miR-29b-3p (MIMAT0000100) is known to be associated with both IBD and SI-NET. miR-29b-3p expression level is significantly increased in CD active inflamed tissues and UC serum blood compared to respective control samples (Viennois et al., 2017; Moon et al., 2024); however, it is found downregulated in CD strictured tissue due to its role as an anti-fibrotic regulator (Biancheri et al., 2013). The same study indicates that this downregulation is mediated by TFG-β, and miR-29b upregulation can result in collagen III degradation. Similarly, in SI-NET, miR-29b-3p deficiency was seen in both primary and metastatic samples (Malczewska et al., 2018). Moreover, miR-29b-3p downregulation might also correlate with worse cancer prognosis, though not statistically significant (*p* = 0.1166) (Bowden et al., 2017). Nonetheless, miR-29b-3p dysfunction confirmed its regulatory mechanism in promoting ECM accumulation, the innermost significant pathway shared between IBD and SI-NET in this study. Lastly, while we identified YY1 to be the most significant TF regulating both upregulated hub genes shared between CD, UC, and SI-NET, its particular role in the pathogenesis of both diseases remains largely unknown.

There are several limitations in our study. The main limitation is that the microarray expression data were analyzed only through computational and statistical approaches and lacked laboratory experimental validations. While we tried to compensate for such limitation with extensive literary review, several of the biological pathways (e.g., the role of TLR9-expressing dendritic cells and anergic T cells) and regulatory molecules (e.g., YY1) found in our study have also not been documented elsewhere. Additionally, we used relatively small sample sizes, particularly in the UC and SI-NET cohorts. Lastly, the SI-NET samples were extracted from patients undergoing Somatostatin analog treatment, which could possibly interfere with the transcriptomic expression level analyzed in this study. Henceforth, further studies are needed to validate and uncover more molecular signatures shared between IBD and SI-NET.

## 5 Conclusion

We report for the first time, to the best of our knowledge, the molecular relationships between IBD and SI-NET. Our Jaccard similarity index suggests overlaps that is comparably as strong as the relationship between IBD and colorectal cancer. Our systems biology analysis indicates TIMP1-mediated myofibroblast activation and intestinal fibrosis as key dysregulated pathways in both diseases, interacting bidirectionally with fatty acid and glucose metabolic reprogramming, namely the Warburg effect. Therefore, future clinical studies should further elucidate the role of these processes as to validate the pathways in which chronic inflammation induces carcinogenesis. Moreover, reversal of these dysregulated pathways (e.g., inhibit aerobic glycolysis and promote oxidative phosphorylation to counteract the Warburg effect) are potential therapeutic approaches to mitigate the pathological damages of IBD and SI-NET. We also identified regulatory mechanisms specific to each IBD subtype that are potential therapeutic targets: in CD and SI-NET, IL-6 and ciliary-dependent signaling pathways exacerbates fibrosis, and in UC and SI-NET, macrophages and the NCAM signaling pathway, though less extensively studied, also promote ECM accumulation. Finally, we documented 17 central genes, 7 protein complexes, and 12 regulatory molecules that can both serve as candidate markers and therapeutic targets for both diseases.

## Data Availability

The original raw data files analyzed in this study are documented in the Gene Expression Omnibus database with accession numbers GSE75214, GSE36807, and GSE65286.
